# Multidrug-resistant phenotypes of genetically diverse *Escherichia coli* isolates from healthy domestic cats

**DOI:** 10.1038/s41598-024-62037-8

**Published:** 2024-05-17

**Authors:** Virginia Núñez-Samudio, Gumercindo Pimentel-Peralta, Alexis De La Cruz, Iván Landires

**Affiliations:** 1 Instituto de Ciencias Médicas, PO Box 0710-00043, Las Tablas, Los Santos Panama; 2 Sección de Epidemiología, Departamento de Salud Pública, Región de Salud de Herrera, Ministry of Health, Chitré, Herrera Panama; 3 Laboratorio de Calidad de Agua, Ministry of Health, Chitré, Herrera Panama; 4 Hospital Regional Dr. Joaquín Pablo Franco Sayas, Región de Salud de Los Santos, Ministry of Health, Las Tablas, Los Santos Panama

**Keywords:** Extended spectrum beta-lactamase, Domestic cats, Epidemiology, Antimicrobial resistance, Panama, *Escherichia coli*, Microbiology, Antimicrobials, Bacteria

## Abstract

Β-lactamases-producing *Escherichia coli* are a widely distributed source of antimicrobial resistance (AMR), for animals and humans. Little is known about the sensitivity profile and genetic characteristics of *E. coli* strains isolated from domestic cats. We report a cross-sectional study that evaluated *E. coli* strains isolated from domestic cats in Panama. For this study the following antibiotics were analyzed: ampicillin, amoxicillin-clavulanate cefepime, cefotaxime, cefoxitin, ceftazidime, aztreonam, imipenem, gentamicin, kanamycin, streptomycin, tetracycline, ciprofloxacin, nalidixic acid, trimethoprim-sulfamethoxazole, and chloramphenicol. The data obtained were classified as resistant, intermediate, or sensitive. MDR strains were established when the strain presented resistance to at least one antibiotic from three or more antimicrobial classes. Forty-eight *E. coli* isolates were obtained, of which 80% presented resistance to at least one of the antibiotics analyzed, while only 20% were sensitive to all (p = 0.0001). The most common resistance was to gentamicin (58%). Twenty-nine percent were identified as multidrug-resistant isolates and 4% with extended spectrum beta-lactamase phenotype. The genes *bla*TEM (39%), *bla*MOX(16%), *bla*ACC (16%) and *bla*EBC (8%) were detected. Plasmid-mediated resistance *qnr*B (25%) and *qnr*A (13%) are reported. The most frequent sequence types (STs) being ST399 and we reported 5 new STs. Our results suggest that in intestinal strains of *E. coli* isolated from domestic cats there is a high frequency of AMR.

## Introduction

*Escherichia coli* is among the main bacteria responsible for infections in both humans and animals. It is one of the key pathogens contributing to the burden of antimicrobial resistance (AMR) and resistance-associated deaths^1^. While some pathogenic strains of *E. coli* can cause intestinal infections, the majority of strains can potentially cause extra-intestinal infections, mainly of the urinary tract^2^. Based on distinctive genotypes, the main phylogenetic groups of *E. coli* have been established (A, B1, B2, and D). The pathogenic extra-intestinal strains belong mainly to Group B2 and, to a lesser extent, to Group D, while the strains of Groups A and B1 are usually intestinal pathogens or commensals^3^.

*Escherichia coli* infections are widely treated with β-lactam antibiotics and fluoroquinolones in both human and veterinary medicine. A study conducted in Latin America reported the antibiotics most used in this region for the treatment of infections in pets corresponded to β-lactams, prescribed in 65.3% and quinolones in 36.2% of the cases treated^4^. However, resistance to these groups of antibiotics has increased worldwide, which is why the World Health Organization (WHO) has devised a list of priority pathogens to combat AMR, where multidrug-resistant (MDR) *E. coli* made the list as a critical priority 1 pathogen^5^.

Among *E. coli* strains, the main mechanism of AMR to β-lactams is the production of β-lactamase enzymes, among which are the extended-spectrum β-lactamase (ESBL) enzymes and the AmpC type encoded by plasmids (pAmpC). ESBL/AmpC–producing *E. coli* constitutes a widely distributed source of AMR in both humans and animals^6^. On the other hand, within the mechanisms of AMR to quinolones, chromosomal amino acid substitutions have been mainly described within the quinolone resistance determining regions (QRDR) of the genes *gyrA* and *parC*, which are targets of quinolones, in addition to the acquisition of plasmid-mediated quinolone resistance (PMQR) genes, among which the *qnr* genes (*qnrA*, *qnrB*, *qnrC*, *qnrD*, and *qnrS*) have been described^7^.

Transmission of *E. coli* with ESBL/AmpC has become a One Health issue, as these strains can be transmitted between humans, animals, and the environment. Close contact between companion animals and humans increases the risk of transmission of ESBL/AmpC-carrying bacteria through horizontal transfer and clonal spread^8,9^. Worldwide, around 56% of people have at least one pet, with domestic cats (*Felis catus*) among the most popular in the world. For example, in a survey conducted in the United States (US), 45.3 million households reported having at least one cat^10^, reflecting the magnitude of its presence in domestic cats.

Healthy cats are recognized as a reservoir of *E. coli* strains carrying ESBL/AmpC, with a global prevalence of 5.04%, predominantly identifying the CTX-M type for ESBL and CMY for pAmpC^11^. Studies carried out in Latin America (i.e., in Argentina and Brazil) on *E. coli* isolates obtained from cats have detected ESBL of the CTX-M-15 and CTX-M-2 types and for the plasmid type pAmpC, the CMY-2 has been reported^12,13^.

In both pets and humans, quinolone resistance has been most frequently reported in β-lactam–resistant Enterobacteriaceae. Studies have demonstrated the association between genes encoding ESBL and gene variants encoding PMQR, such as *qnrA*, *qnrB*, and *qnrS* genes^14,15^.

In companion animals, around 171 different sequence types (STs) of *E. coli* have been reported, with ST38 and ST131 found in all continents^11^. In Latin America, ST90, ST457, ST155, and ST131 have been predominantly identified in cat samples^14,15^.

Studies carried out in Latin America, specifically in South America, show a high percentage of resistance to multiple antibiotics in strains of *E. coli* isolated from cats^14,15^. However, as far as we know, there are no reports published for Central America. The purpose of this study is, then, to characterize *E. coli* strains isolated from fecal samples of domestic cats in the central region of Panama, with the objective of investigating the AMR phenotype by identifying sensitivity profiles, characterizing the ESBL/AmpC–producing strains and their molecular typing using the multilocus sequence typing (MLST) scheme and phylogenetic group analysis.

## Results

### *Escherichia coli* strains isolated from fecal samples of domestic cats

A total of 48 *E. coli* strains were isolated from domestic cats in the central region of Panama, of which 75% (36/48) were obtained from females and 25% (12/48) from males, with a mean (SD) age of 13.2 (14.5) months. The cat breeds were all undefined and the type of feeding was distributed in 56% (27/48) for kibbles, 40% (19/48) varied and 4% (2/48) a mixture of varied and kibbles. Ten percent (5/48) had a history of previous antibiotic use, including: penicillin (2/5), metronidazole/spiramycin (1/5), amoxicillin (1/5), and enrofloxacin (1/5).

### Sensitivity profile of *E. coli* strains

**Figure 1:**
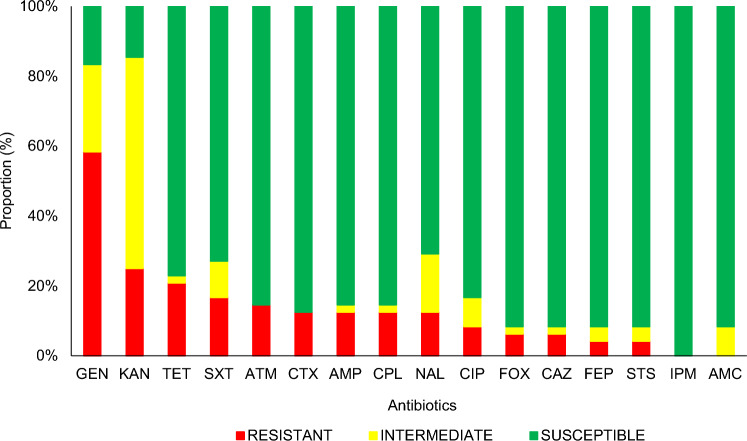
Sensitivity profile of *E. coli* strains isolated from domestic cats in central Panama. AMC, amoxicillin–clavulanate; AMP, ampicillin; ATM, aztreonam; CAZ, ceftazidime; CIP, ciprofloxacin; CPL, chloramphenicol; CTX, cefotaxime; FEP, cefepime; FOX, cefoxitin; GEN, gentamicin; IPM, imipenem; KAN, kanamycin; NAL, nalidixic acid; STS, streptomycin; SXT, trimethoprim–sulfamethoxazole; TET, tetracycline.

Of the *E. coli* strains analyzed, 80% (38/48) showed resistance to at least one of the antibiotics and 20% (10/48) were sensitive to all antibiotics analyzed (*P* < 0.001). Figure 1 presents the sensitivity to the antibiotics analyzed with the *E. coli* isolates. We note greater resistance to gentamicin (58%), kanamycin (25%), and tetracycline (21%) and no resistance to amoxicillin-clavulanate and imipenem. No significant differences were evident when comparing the proportion of resistance by age, sex, breed, type of diet, and history of previous antibiotic use.

### Proportion of antimicrobial resistance in the identified phylogenetic groups

Table 1 shows the phylogeny classification of the isolated *E. coli* strains, with 65% commensals from phylogenetic groups A or B1 and 33% pathogenic from phylogenetic group B2; no strains from phylogenetic Group D were identified. Regarding the distribution of isolates with antibiotic resistance by phylogroup, Table 2 shows that gentamicin was the antibiotic presenting the highest proportion of resistance in the three phylogenetic groups: for Group A, 33% of the strains presented resistance to three or more antibiotics, for Groups B1 and B2, respectively, 30% and 38% presented resistance to three or more antibiotics, with no significant difference between phylogenetic groups (*P* = 0.182).

**Table 1:** Phylogenetic analysis of *E. coli* strains isolated from domestic cats in central Panama.

*chuA*	*yjaA*	*TSPE4.C2*	Phylogenetic group	*n* (%)
−	+	−	A	12 (25)
−	+	+	B1	20 (42)
+	+	+	B2	14 (29)
+	+	−	B2	2 (4)
+	−	+	D	0 (0)

**Table 2:** Phylogenetic analysis of *E. coli* strains isolated from domestic cats in central Panama.

Antibiotic	Phylogenetic group, n (%)		
	A (n = 12)	B1 (n = 20)	B2 (n = 16)
AMP	2 (17)	4 (20)	–
ATM	2 (17)	2 (10)	3 (19)
FOX	1 (8)	–	2 (13)
CAZ	1 (8)	1 (5)	1 (6)
CPL	2 (17)	2 (10)	2 (13)
CTX	1 (8)	1 (5)	3 (19)
FEP	–	1 (5)	1 (6)
CIP	1 (8)	2 (10)	–
NAL	3 (25)	1 (5)	2 (13)
STS	1 (8)	1 (5)	–
SXT	2 (17)	3 (15)	2 (13)
TET	3 (25)	4 (20)	2 (13)
GEN	8 (67)	13 (65)	7 (44)
KAN	4 (33)	7 (35)	1 (6)

AMP, ampicillin; ATM, aztreonam; FOX, cefoxitin; CAZ, ceftazidime; CPL, chloramphenicol; CTX, cefotaxime; FEP, cefepime; CIP, ciprofloxacin; NAL, nalidixic acid; STS, streptomycin; SXT, trimethoprim-sulfamethoxazole; TET, tetracycline; GEN, gentamicin; KAN, kanamycin.

### Phenotypes and genotypes

Molecular typing using Pasteur's MLST technique identified 18 STs (Table 3). We observed ST399 (17%), ST1242 (17%), ST1243 (9%), and 1% for each of the remaining STs. Five 5 new STs are reported: ST1242, ST1243, ST1244, ST1245, and ST1247.

**Table 3:** Phenotypes and genotypes of *E. coli* strains isolated from domestic cats in central Panama.

Isolate	Phylo-group	ST Pasteur	ST Achtman	β-lactamase	Antimicrobial resistance
GLS08^a,b^	A	1243	48	TEM	AMP, ATM, CPL, GEN, SXT, TET
GLS09^a,b^	A	1243	48		ATM, CAZ, CTX, KAN, NAL, SXT
GLS14	A	532	1684		GEN, KAN
GLS24^a^	A	367	10	EBC	CTX, FOX, GEN, NAL, STS
GLS25^a^	A	132	10		CIP, GEN, KAN, NAL, TET
GLS26	A	ND	ND		GEN
GLS28	A	ND	ND		GEN
GLS29^a^	A	88			AMP, CPL, GEN, KAN, SXT, TET
GLS35	A	ND	ND		GEN
GLS36	A	ND	ND		
GLS37	A	ND	ND		
GLS42	A	ND	ND		
GLS02	B1	355			ATM, CIP
GLS03	B1	ND	ND		GEN
GLS06	B1	ND	ND		GEN
GLS07^a^	B1	ND	ND	TEM	AMP, CIP, CPL, GEN, KAN, NAL, SXT, TET
GLS18^a^	B1	399	2144/2178	TEM	AMP, GEN, KAN, TET
GLS20	B1	481	21		KAN
GLS21^b^	B1	1245			GEN, KAN
GLS22^a^	B1	399	2144/2178	TEM	AMP, GEN, STS, TET
GLS27	B1	501	677		GEN, KAN
GLS30	B1	ND	ND		GEN
GLS33^a^	B1	ND	ND	TEM	AMP, CPL, GEN, KAN, SXT, TET
GLS34	B1	83			CIP, KAN
GLS38	B1	ND	ND		GEN
GLS39^b^	B1	1247			GEN
GLS40	B1	ND	ND		GEN
GLS43^a,c^	B1	529	2016		ATM, CAZ, CTX, FEP, SXT
GLS44	B1	ND	ND		
GLS47	B1	955	336		GEN
GLS48	B1	ND	ND		GEN
GLS49	B1	ND	ND		
GLS01^a,b^	B2	1242	929	MOX, ACC	ATM, CPL, CTX, FOX, TET
GLS05	B2	399	2144/2178		KAN, NAL
GLS10^†^	B2	1242	929		GEN
GLS11	B2	ND	ND		GEN
GLS12^a,b^	B2	1242	929		GEN, NAL, SXT
GLS13	B2	86			
GLS15	B2	ND	ND		GEN
GLS16	B2	218			GEN, TET
GLS17^a,b^	B2	1244	6355	MOX, ACC	ATM, CPL, CTX, FOX
GLS19	B2	ND	ND		
GLS31	B2	ND	ND		
GLS32	B2	ND	ND		GEN
GLS41^a,c^	B2	ND	ND		ATM, CAZ, CTX, FEP, SXT
GLS45	B2	ND	ND		
GLS46	B2	ND	ND		GEN
GLS50	B2	ND	ND		

AMC, amoxicillin-clavulanate; AMP, ampicillin; ATM, aztreonam; CAZ, ceftazidime; CIP, ciprofloxacin; CTX, cefotaxime; FEP, cefepime; CPL, chloramphenicol; FOX, cefoxitin; IPM, imipenem; GEN, gentamicin; KAN, kanamycin; NAL, nalidixic acid; ND, not determined; STS, streptomycin; SXT, trimethoprim-sulfamethoxazole; TET, tetracycline.

^a^Multidrug resistant (MDR).

^b^New sequence typing (ST).

^c^Extended-spectrum beta-lactamase (ESBL).

The characterization of MDR strains was detected in 29% (14/48), among which 36% (5/14) presented resistance to three, 36% (5/14) to four, and 28% (4/14) to five or more types of antibiotics. We identified that 27% (13/48) of the strains were resistant to at least one of the β-lactams analyzed and 4% (2/48) presented the ESBL phenotype. Regarding the genotypes of the strains showing β-lactam resistance, the *bla*TEM gene was identified in 39% (5/13), while *bla*CTX-M and *bla*SHV were not detected. Within *pAmpC* genes, *bla*MOX was identified in 16% (2/13), *bla*ACC in 16% (2/13), and *bla*EBC in 8% (1/13).

Table 4 shows the PMQR and QRDR for the strains showing quinolone and fluoroquinolone resistance, with 38% (3/8) showing changes in *gyrA* and ST355 (GLS02) with changes in *parC*. We observed the following changes: Ser83Leu (38%, 3/8), Asp87Asn (13%, 1/8), and Ser80Ile (13%, 1/8). Among the strains with quinolone resistance, we identified *qnrA* genes in 13% (1/8) and *qnrB* in 25% (2/8).

**Table 4:** Distribution by ST of QRDR and PMQR genes in *E. coli* isolates resistant to quinolones.

Strain	ST Pasteur	PMQR	QRDR	*gyrA*		*parC*		Quinolone resistance
				83	87	80	84	
*E. coli* K12				Ser (TCG)	Asp (GAC)	Ser (AGC)	Glu (GAA)	
GLS_02	355		3	**Leu (TTG)**^a^	**Asn (AAC)**^a^	**Ile (ATC)**^a^	Glu (GAA)	CIP
GLS_05	399		0	Ser (TCG)	Asp (GAC)	Ser (AGC)	Glu (GAA)	NAL
GLS_07	ND		1	**Leu (TTG)**^a^	Asp (GAC)	Ser (AGC)	Glu (GAA)	CIP, NAL
GLS_09	1243		0	Ser (TCG)	Asp (GAC)	Ser (AGC)	Glu (GAA)	NAL
GLS_12	1242		0	Ser (TCG)	Asp (GAC)	Ser (AGT)	Glu (GAA)	NAL
GLS_24	367		0	Ser (TCG)	Asp (GAC)	Ser (AGC)	Glu (GAA)	NAL
GLS_25	132	*qnrB*	1	**Leu (TTG)**^a^	Asp (GAC)	Ser (AGC)	Glu (GAA)	CIP, NAL
GLS_34	83	*qnrA, qnrB*	0	Ser (TCG)	Asp (GAC)	Ser (AGC)	Glu (GAA)	CIP

Asn, asparagine; CIP, ciprofloxacin; Glu, glutamine; Ile, isoleucine; Leu, leucine; NAL, nalidixic acid; ND, not determined; PMQR, plasmid mediated quinolone resistance; QRDR, quinolone resistance determining region; Ser, serine; ST, sequence typing.

Significant values are in bold.

^a^Substitutions.

## Discussion

*Escherichia coli* with ESBL has become a One Health problem, since it can be transmitted among humans, animals, and the environment and represents one of the main pathogens contributing to the AMR burden and resistance-associated mortality^1^. The present study shows bacterial phenotypes in combination with genetic characteristics of *E. coli* strains isolated from healthy domestic cats from the central region of Panama. We observed that 80% of the *E. coli* strains presented resistance to at least one of the antibiotics analyzed, among which we identified the ESBL phenotype in 4%. Worldwide, it has been estimated that the prevalence of ESBL in cats is 5.04% and in dogs it is 6.87%^11^. This prevalence may vary by region, for example, in America it has been reported in cats 8.15% and in dogs 6.79%^11^. Therefore, the ESBL prevalence identified in our study concurs with region’s reported prevalence rates. Globally, no significant differences have been observed between the prevalence of ESBL between dogs and cats. Similarly in Panama, a previous study conducted on *E. coli* strains isolated from healthy dogs identified an 8% ESBL phenotype^16^. Worldwide, the most identified ESBL genotypes are *bla*CTX-M, *bla*SHV, and *bla*TEM, which are uniformly distributed between dogs and cats. However, the prevalence rates of these phenotypes may vary between regions^11^. In our study, the *bla*TEM , *bla*CTX-M and *bla*SHV were not identified among the ESBL strains tested. These findings are not in agreement with data from studies conducted in Latin America where ESBL of the CTX-M-2 and CTX-M-15 genotypes have been identified in *E. coli* strains isolated from cats^12,13^.

Different groups of pAmpC enzymes have been described, including: CMY, ACC, DHA, CIT, EBC, FOX, and MOX, which have been detected in both humans and animals^17^. The CMY-2 type is the pAmpC most identified in domestic animals. However, in our study, pAmpC of the MOX, ACC, and EBC were the main groups identified^8^. Previous studies carried out on *E. coli* strains isolated from cats have identified pAmpC of the EBC and ACC groups^17^, however the report of MOX group enzymes in companion animals is unusual—there are reports in production animals, such as chickens and goats^18,19^. In our study, two strains (GLS01 and GLS17) co-expressed the *bla*ACC and *bla*MOX genes, which shows an exchange of *E. coli* strains carrying important interspecies resistance genes, since the MOX group has been described mainly in production animals. This represents an important source of MDR dissemination, given that encoding genes are carried in mobile elements and confer resistance to broad-spectrum cephalosporins.

Of the strains analyzed in this study, 29% were MDR within all phylogenetic groups (A, B1, and B2) with no significant differences between groups. This finding is of particular concern, due to the potential for opportunistic infections, zoonotic transmission, and transmission of antibiotic-resistant determinants from commensal isolates to potential pathogenic bacteria^20^. MDR *E. coli* strains have been previously described in studies carried out in healthy domestic animals^11,13–17^ as well as from environmental strains^21^. This aspect has varied in diverse countries where regulatory standards based on non-prescription antibiotics used in veterinary medicine have been regulated due to the emergence of MDR strains^22^. On the other hand, aspects such as diet, use of antibiotics without volumetric determination, commonly used antibiotics (e.g., doxycycline and enrofloxacin) all contribute to the emergence of resistant strains^23^. MDR *E. coli* poses a challenge to global health systems due to the treatment of infectious diseases and increased therapeutic complications^24^.

Around 171 different STs have been described in cats and dogs, with ST38 and ST131 identified on all continents, followed by ST68, ST405, and ST617^11^. In Latin America, the prevalence of STs obtained by the MLST technique from *E. coli* strains isolated in cats varies between countries. For example, in Brazil and Argentina, ST90, ST457, and ST131 have been identified as the most predominant^12,13^. In our study, ST399 was the most prevalent and interestingly, this same ST was mostly identified in a study of *E. coli* strains isolated from healthy dogs in central Panama^16^, which could be due to the close coexistence of dogs and cats in the same households. Likewise, ST399 has been described in isolates from dogs and cats in Oceania and Asia^11^. In addition, ST532, previously described in Europe, and ST367 and ST132—described in the Americas, Asia, and Europe^11^ were identified. The above evidences the existence of an important distribution of circulating genetic diversity.

The ST88 identified in this study has been previously reported in clinical isolates from hospitalized patients in our country^25^ and also in isolates from healthy dogs^16^, which allows us to infer that there may be exchange of *E. coli* strains carrying genes of resistance between owners, pets, and the environment^26^. ST132 and ST83 presented the expression of PMQR genes, with ST83 presenting co-expression of resistance genes *qnrB* and *qnrS*. As stated, it is paramount that pet owners and their relatives may be at risk of acquiring bacteria carrying antibiotic resistance genes and plasmid-mediated genes. A study showed that the transport of community-acquired ESBL/pAmpC was attributed in 7.9% to companion animals, constituting the most common nonhuman sources^6^.

The WHO establishes among its critically important antibiotics the quinolones and β-lactams (including 3rd, 4th, and 5th generation cephalosporins and amino penicillins with and without beta-lactamase inhibitors) in addition to aminoglycosides and others^27^. Many of these are also used in veterinary medicine. Our data showed resistance to several of the aforementioned critically important antibiotics (Fig. 1), which draws attention to the potential difficulties in managing infections, as well as the importance of knowing the composition and distribution of antibiotic resistance genotype as an important step to delineate the impact of MDR *E. coli* infections in our population.

The limitations of this study have to do with the small number of strains analyzed due to the high cost of inputs in Panama. Limitations aside and to the best of our knowledge, this is the first study in Panama and Central America on patterns of antimicrobial sensitivity and resistance, as well as molecular epidemiology data of *E. coli* strains isolated from fecal samples of domestic cats showing MDR is important in commensal strains of *E. coli* in healthy cats widely distributed among other carrier species, some of them of clinical interest, as well as the new STs described for *E. coli*.

## Methods

### Strain isolates

A cross-sectional study was conducted in August 2022, where fecal samples were obtained from domestic cats from the central provinces of Herrera and Los Santos in the Republic of Panama, with the informed consent of their owners. Samples were obtained by rectal swabbing of domestic cats in Cary-Blair media (COPAN Diagnostics Inc.; Murrieta, CA) for *E. coli* isolates. For each sample collected, a technical sheet was completed with the following variables, if present: age, sex, breed, diet, and previous use of antibiotics. One sample was obtained per individual. *E. coli* isolates were obtained using Chromocult^®^ agar (Merck Millipore; Darmstadt, Germany) and on eosin-methylene blue (EMB) agar (Merck Millipore; Darmstadt, Germany) for confirmation. The study protocol was reviewed and approved by the Animal Bioethics Subcommittee at the University of Panama’s Committee of Ethics of Research and Animal Welfare (No. CEIBA-UP-027-2022, dated 11 July 2022), all methods were performed in accordance with the relevant guidelines and regulations.

### Antibiotic sensitivity testing

Antibiotic sensitivity was determined by the disk diffusion method on Muller Hinton Agar (Merck Millipore; Darmstadt, Germany)^28^. Sensitivity profiles were interpreted according to the Clinical and Laboratory Standards Institute^29^. The following antibiotics were analyzed: ampicillin (AMP 10 μg), amoxicillin-clavulanate (AMC 20/10 μg), cefepime (FEP 30 μg), cefotaxime (CTX 30 μg), cefoxitin (FOX 30 μg), ceftazidime (CAZ 30 μg ), aztreonam (ATM 30 µg), imipenem (IPM 10 µg), gentamicin (GEN 10 µg), kanamycin (KAN 30 µg), streptomycin (STS 300 µg), tetracycline (TET 30 µg), ciprofloxacin (CIP 5 µg) , nalidixic acid (NAL 30 µg), trimethoprim-sulfamethoxazole (SXT 1.25/23.75 µg), and chloramphenicol (CPL 30 µg). The data obtained were classified as resistant, intermediate, or sensitive. MDR strains were established when the strain presented resistance to at least one antibiotic from three or more antimicrobial classes^30^.

### Molecular typing

Molecular typing using the MLST technique was conducted on the strains showing resistance to more than one antibiotic and four strains with resistance to one antibiotic were chosen at random (GLS20, GLS39, GLS47, and GLS10) in addition to a strain sensitive to all antibiotics (GLS13). DNA extraction from the isolates was carried out using the boiling method. The extracted DNA was stored at − 20 °C to perform molecular tests. The Pasteur Institute MLST technique was used to study eight genes: *dinB*, *icdA*, *polB*, *pabB*, *putP*, *trpA*, *trpB*, and *uidA*^31^. Samples were amplified from chromosomal DNA, analyzed by polymerase chain reaction (PCR) in a MiniAmp Plus Thermal Cycler (Applied Biosystems; Waltham, MA) and visualized in electrophoresis with 1.5% agarose gel (Thermo Fisher Scientific; Waltham, MA). Sequencing of PCR products was performed using Macrogen services (Macrogen Inc.; Seoul, Korea). The sequences of the eight genes were analyzed in Geneious Prime v.2020 2.4. (Dotmatics; Boston, MA) and were run on the Institut Pasteur MLST Bacterial Isolate Genome Sequence Database (BIGSDB) website for particular allele profiles and STs (https://bigsdb.pasteur.fr/ecoli/ecoli.html). A search was conducted for the STs identified in our study and the corresponding STs using Achtman’s MLST Method (https://pubmlst.org/bigsdb?db=pubmlst_escherichia_seqdef).

### Amplification of resistance genes

The isolates based on the resistance phenotype to β-lactams and quinolones were analyzed by PCR to determine the presence of *bla*CTX-M, *bla*TEM, *bla*SHV, *bla*AMPC genes, using specific PCR primers, as previously described and summarized in Table 5^16,31–33^. The fragments obtained from the PCR product were analyzed by electrophoresis in a 1.5% agarose gel and visualized under ultraviolet light. The presence of *qnr*A, *qnr*B, and *qnr*S was determined for PMQR and QRDR with *gyrA* and *parC*^34,35^. The results obtained from the sequencing of *gyrA* and *parC* were analyzed in Geneious Prime v.2020 2.4, BLASTN nucleotide search program of the National Center for Biotechnology Information (NCBI) (https://blast.ncbi.nlm.nih.gov/Blast.cgi) and compared with partial sequences of *E. coli* K12 (X57174.1, M58408.1).

**Table 5:** Primers used in the study.

Target	Primer sequence (5ʹ–3ʹ)	Ta (ºC)	Product (bp)	References
CTX-M-F	ATGTGCAGYACCAGTAARGTKATGGC	55	592	^33^
CTX-M-R	TGGGTRAARTARGTSACCAGAAYSAGCGG			
TEM-F	GCGGAACCCCTATTTG	55	964	^34^
TEM-R	ACCAATGCTTAATCAGTGAG			
SHV-F	TTATCTCCCTGTTAGCCACC	55	796	^35^
SHV-R	GATTTGCTGATTTCGCTCGG			
CMY-F	ATGATGAAAAATCGTTATGCTGC	58	1138	^34^
CMY-R	GCTTTTCAAGAATGCGCCAGG			
AmpC1-71-F	AATGGGTTTTCTACGGTCTG	55	191	^34^
AmpC2120-R	GGGCAGCAAATGTGGAGCAA			
gyrA-F	AAATCTGCCCGTGTCGTTGGT	58	344	^36^
gyrA-R	GCCATACCTACGGCGATACC			
parC-F	CTGAATGCCAGCGCCAAATT	57	168	^36^
parC-R	GCGAACGATTTCGGATCGTC			
qnrA-F	ATTTCTCACGCCAGGATTTG	53	516	^35^
qnrA-R	GATCGGCAAAGGTTAGGTCA			
qnrB-F	GATCGTGAAAGCCAGAAAGG	53	469	^35^
qnrB-R	ACGATGCCTGGTAGTTGTCC			
qnrS-F	ACGACATTCGTCAACTGCAA	53	417	^35^
qnrS-R	TAAATTGGCACCCTGTAGGC			
ACC-F	CACCTCCAGCGACTTGTTAC	60	346	^32^
ACC-R	GTTAGCCAGCATCACGATCC			
CIT-F	CGAAGAGGCAATGACCAGAC	60	538	^32^
CIT-R	ACGGACAGGGTTAGGATAGY			
DHA-F	TGATGGCACAGCAGGATATTC	60	997	^32^
DHA-R	GCTTTGACTCTTTCGGTATTCG			
EBC-F	CGGTAAAGCCGATGTTGCG	60	683	^32^
EBC-R	AGCCTAACCCCTGATACA			
FOX-F	CTACAGTGCGGGTGGTTT	60	162	^32^
FOX-R	CTATTTGCGGCCAGGTGA			
MOX-F	GCAACAACGACAATCCATCCT	60	895	^32^
MOX-R	GGGATAGGCGTAACTCTCCAA			
VEB-F	CATTTCCCGATGCAAAGCGT	60	648	^32^
VEB-R	CGAAGTTTCTTTGGACTCTG			

F: forward; R: reverse; bp: base pairs; Ta: temperature.

### Phylogenetic analysis

The distribution of phylogenetic groups of commensal and pathogenic strains was carried out based on methodologies described by Clermont’s group^3^. The fragments obtained were analyzed by electrophoresis in 2% agarose gel and visualized under ultraviolet light.

### Data analyses

Data were recorded in MS Excel (The Microsoft Corporation; Redmond, WA, US). Data analysis was performed in Stata v. 11.0 (StataCorp, LLC; College Station, TX). We applied descriptive statistics and estimated their respective 95% confidence intervals (CIs). We used Fisher’s exact test to compare proportions. An alpha at 0.05 was set for a significant statistic to compare antibiotic resistance frequencies by age, sex, and previous antibiotic group use.

### Institutional review board statement

The study protocol was reviewed and approved by the Animal Bioethics Committee at the University of Panama: Comité de Ética de la Investigación y el Bienestar de los Animales de la Universidad de Panamá CEIBAUP (No. CEIBA-UP-027-2022, dated 11 July 2022).

### Informed consent statement

Informed consent was obtained from all owners of cats participating in the study.

### Use of generative artificial intelligence (AI)

The authors declare that no generative AI tools or services were used in the preparation of this manuscript.

## Data Availability

All data generated or analysed during this study are included in this published article. The study is reported in accordance with ARRIVE guidelines.
